# 3D spheroid culture enhances survival and therapeutic capacities of MSCs injected into ischemic kidney

**DOI:** 10.1111/jcmm.12651

**Published:** 2016-02-24

**Authors:** Yong Xu, Taoping Shi, Axiang Xu, Lei Zhang

**Affiliations:** ^1^ Department of UrologyPLA General HospitalHaidian DistrictBeijingChina

**Keywords:** acute kidney injury, mesenchymal stem cells, paracrine secretion, three‐dimensional culture

## Abstract

Three‐dimensional (3D) cell culture has been reported to increase the therapeutic potentials of mesenchymal stem cells (MSCs). In this study, we aimed to investigate the therapeutic effects of 3D spheroids of human adipose‐derived MSCs for acute kidney injury (AKI). *In vitro* studies indicated that 3D spheroids of MSCs produced higher levels of extracellular matrix proteins (including collagen I, fibronectin and laminin), and exhibited stronger anti‐apoptotic and anti‐oxidative capacities than two‐dimensional (2D) cultured cells. Furthermore, 3D culture increased the paracrine secretion of cytokines by MSCs, including angiogenic factors (VEGF and basic fibroblast growth factor), anti‐apoptotic factors (epidermal growth factor and hepatocyte growth factor), the anti‐oxidative factor insulin‐like growth factor and the anti‐inflammatory protein tumour necrosis factor‐alpha stimulated gene/protein 6. Consistent with *in vitro* experiments, 3D spheroids of MSCs showed enhanced survival and paracrine effects *in vivo*. More importantly, when injected into the kidney of model rats with ischemia‐reperfusion (I/R)‐induced AKI, 3D spheroids were more beneficial in protecting the I/R kidney against apoptosis, reducing tissue damage, promoting vascularization and ameliorating renal function compared with 2D cultured cells. Therefore, the 3D culture strategy improved the therapeutic effects of MSCs, and might be promising for AKI treatment.

## Introduction

Acute kidney injury (AKI) is a clinical syndrome characterized by a sudden decline in kidney function 1, and affects up to 7% of hospitalized patients 2. The causes of AKI are numerous, including renal ischemia, exposure of the kidney to harmful substances and inflammatory process 3. Despite increasing studies in the past few decades, AKI continues to be associated with high mortality rates from 15% to 60%, without efficient treatment methods at present 4.

Stem cell‐based therapy is regarded as a promising strategy for AKI treatment. Different types of stem cells, including mesenchymal stem cells (MSCs) 5, 6, embryonic stem cells 7, amniotic fluid stem cells 8, and hematopoietic stem and progenitor cells 9, have been investigated in their therapeutic potentials for AKI. Among these stem cells, MSCs were studied most extensively due to their ease of isolation, abundant distribution and low immunogenicity. Recent studies indicated that transplantation of MSCs prevented and repaired damage to the kidney during AKI induced by cisplatin 5, 10, 11, 12, or ischemia‐reperfusion (I/R) 6, 13, 14, 15.

Mesenchymal stem cells have the self‐renewal potential and can differentiate into multiple cell types, such as osteoblasts, chondrocytes and adipocytes. Although it was originally supposed that injected MSCs might differentiate to replace injured cells, therapeutic effects were frequently observed without engraftment and differentiation of donor cells 13, 16. Instead, paracrine secretion acted as an important mechanism for stem cell‐based tissue repair. Cytokines produced by MSCs exhibited multiple beneficial functions, including promoting angiogenesis, inhibiting apoptosis, reducing inflammation and scavenging reactive oxygen species (ROS) 17, 18. However, paracrine secretion is limited under normal culture conditions, and many MSCs transplanted into in ischemic tissues may die of anoikis before they release cytokines. Therefore, the therapeutic benefits of MSCs were severely impaired. To improve the therapeutic potentials of MSCs, several strategies have been developed, such as hypoxia preconditioning 19, 20, 21, 22 and gene modification 16, 23, 24.

Recently, three‐dimensional (3D) culture was reported to promote differentiation of MSCs 25, 26 or to increase their therapeutic potentials 27. Aggregation of MSCs into 3D spheroids enhances the expression of the anti‐inflammatory protein tumour necrosis factor‐alpha stimulated gene/protein 6 (TSG‐6) 28, as well as their paracrine secretion of angiogenic factors, including VEGF, basic fibroblast growth factor (bFGF) and angiogenin 29. Besides, the expression of surface antigens responsible for cell adhesion and motility is substantially changed in 3D spheroids, which is supposed to facilitate their homing in new environments 30. The 3D culture method has been reported to be helpful in the treatment of several diseases, such as peritonitis 28 and myocardial infarction 31. However, it remains unclear whether this strategy also exerts beneficial effects on MSC‐based therapy of AKI.

In this study, we aimed to investigate whether 3D culture of MSCs could have therapeutic benefits for AKI. 3D spheroids of human adipose‐derived MSCs were evaluated for their secretion of extracellular matrix (ECM) proteins and cytokines, as well as their survival under oxidative environments. 2D cultured MSCs or 3D spheroids were transplanted into the kidney of model rats with I/R‐induced AKI. Function, apoptosis, tissue damage and vascularization of ischemic kidneys were determined thereafter.

## Materials and methods

### Isolation, cultivation and characterization of human adipose‐derived MSCs

Adipose tissues were obtained from raw human lipo‐aspirates, and human adipose‐derived MSCs were isolated according to previous studies 32, 33. Briefly, adipose tissues were washed with sterile PBS three times to remove contaminating debris, cut into pieces (<1 mm^3^) with scissors, and then digested with 0.1% collagenase I (Sigma‐Aldrich, St. Louis, Missouri, USA) in PBS. The digestion was performed for 1 hr in 37°C with gentle agitation. After filtering with 80 μm meshes, the solution was centrifuged at 800 × g for 8 min. The pellet was resuspended in fresh medium (α‐MEM/10% FBS) and seeded in tissue culture plates. The plates were placed into an incubator at 37°C with 5% CO_2_. Cells were passaged when they reached 90% confluence, using 0.25% trypsin at a 1:3 split ratio. The immunophenotype of MSCs was analyzed by flow cytometry as previously reported 33. CD105, CD90, CD45 and CD34 (Biolegend, San Diego, California, USA) were analyzed. The multipotency of MSCs was verified by osteogenic and adipogenic differentiation according to previous reports 32.

### Spheroid generation and *in vitro* injection

Hanging drop culture was employed for generation of MSC spheroids according to the previous report 28. Briefly, human adipose‐derived MSCs were prepared as 7.5 × 10^5^/ml cell suspensions. A total of 35 μl of cell solution per drop (containing about 25,000 cells) was prepared onto the covers of culture plate. Cell drops were cultured inversely for 3 days in an incubator at 37°C with 5% CO_2_. To determine the behaviour of cell spheroids after injection, spheroids were collected and injected into culture plates using 1 ml syringes. The growth of conventionally cultured spheroids was observed in culture plates, and functional analysis of spheroids‐derived cells was performed similar to spheroids.

### AKI induction and cell injection

Male adult SD rats were purchased from the Experimental Animal Center, Academy of Military Medical Science (Beijing, China). All the experiments in the study were approved by Animal Care and Use Committee of Chinese PLA General Hospital. Rats were anaesthetized with sodium pentobarbital (30 mg/kg), and the acute renal ischemia/reperfusion (I/R) injury was performed as reported 34. After laparotomy and exposure of the kidney, atraumatic vascular clamps were used to clamp the bilateral renal pedicles. The kidney was subjected to 40 min. of ischemia, followed by reperfusion. The rats of AKI were randomly divided into three groups (*n* = 15/group). At the time of reperfusion, 100 μl of saline or 2 × 10^6^ of 2D cultured MSCs or 3D spheroids (containing 2 × 10^6^ MSCs, spheroids could be dissociated by trypsin/ethylenediaminetetraacetic acid for cell counting) 28 were injected into the kidney cortex using a 1 ml syringes. For *in vivo* tracking, MSCs were pre‐labelled with DiI (1,19‐dioctadecyl‐3,3,3939‐testramethylindocarbocyanine perchlorate; Sigma‐Aldrich) before spheroid formation according to the previous report^35^. After surgery, the abdomen was closed and rats were allowed to recover with cautious care.

### Histological and immunohistochemical analysis

At days 3, 7 and 14 after surgery, the kidneys were obtained and fixed in 4% paraformaldehyde (*n* = 5 for each group). Four micrometre paraffin‐embedded sections were prepared. To determine tissue damage, haematoxylin and eosin staining was performed on paraffin‐embedded sections of days 3 and 7. Tubular injury was evaluated by an experienced technician as reported previously 34. To determine the neovascularization of injured tissues, immunostaining with anti‐vWF (von Willebrand factor, vWF) antibodies (Sigma‐Aldrich) was carried out on paraffin‐embedded sections of days 7 and 14. Positively stained vessels were then identified and quantified under microscope. For each animal, 5 discontinuous sections were observed and 5 random fields were selected on each section. The vascular density was expressed as the number of capillary vessels per field.

### TUNEL staining

Three days after surgery, the kidneys were obtained and fixed in 4% paraformaldehyde (*n* = 5 for each group). Four micrometre paraffin‐embedded sections were prepared. The terminal deoxynucleotidyl transferase dUTP nick‐end labelling (TUNEL) assay was performed using the TUNEL Staining Kit (Invitrogen, Carlsbad, California, USA.) according to the manufacturer's instructions. Positive‐stained cells were counted under microscopy. For each animal, five discontinuous sections were observed, and five random fields were selected on each section.

### Renal function assay

Blood was taken 3 days, 1 week and 2 weeks after the surgery (*n* = 5 for each group). Serum creatinine and blood urea nitrogen (BUN) were measured with a commercial kit (Zhong Sheng Bei Kong Biotechnology Inc., Beijing, China.) according to the manufacturer's instruction 34.

### Western blotting

Cells were collected and lysed in Laemmli Sample Buffer (Bio‐Rad, Hercules, State of California, USA). To extract proteins from tissues, renal samples were homogenized and then lysed in Laemmli Sample Buffer. Proteins were quantified using the BCA^™^ Protein Assay Kit (Thermo Scientific, Waltham, State of Massachusetts, USA). For electrophoresis, 15% SDS‐PAGE was prepared, and 80 μg of total proteins were loaded. After electrophoresis, proteins were transferred to a PVDF membrane (Roche, Basilea, Switherland). The membrane was blocked with 5% non‐fat dried milk (in TBST), and then incubated with primary antibodies at 4°C overnight. Unconjugated antibodies were removed by washing with TBST, and the membrane was further incubated with corresponding horseradish peroxidase‐conjugated secondary antibodies. Protein bands were detected using Enhanced Chemiluminescence Reagent (Applygen, Beijing, China). Band intensities were quantified using a computer‐assisted method and normalized to the internal control. Antibodies for hGAPDH (glyceraldehyde‐3‐phosphate dehydrogenase), collagen I, fibronectin, laminin, p‐Akt, Caspase 3 and Bcl‐2 were purchased from cell signalling technology (Boston, Massachusetts, USA); Antibodies for catalase and superoxide dismutase 1 (SOD‐1) were purchased from Sigma‐Aldrich; hVEGF and pan‐GAPDH were purchased from Abcam; Antibodies for human hepatocyte growth factor (HGF), VEGF, EGF, insulin‐like growth factor (IGF) and bFGF were purchased from R&D Systems China (Shanghai, China.); Anti‐hTSG‐6 antibodies were purchased from Santa Cruz (California, USA). All the antibodies were used in accordance with the instructions provided by the manufacturers.

### Statistics analysis

All data are expressed as mean ± SD. The SPSS 17.0 software (Chicago, USA) was used for statistical analysis. Student's *t*‐test was used for comparison among three groups. A value of *P* < 0.05 was considered statistically significant.

## Results

### Secretion of ECM proteins by human adipose‐derived MSCs under different culture conditions

**Figure 1:**
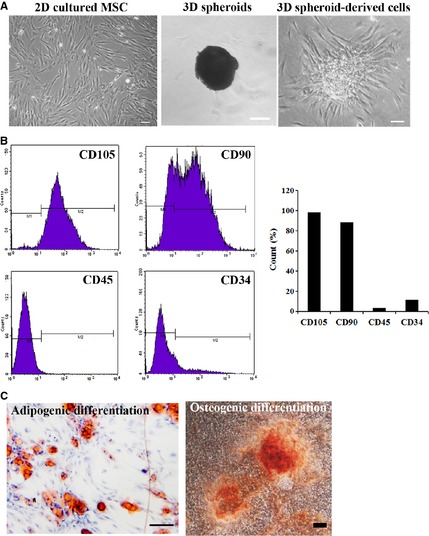
Isolation, cultivation and characterization of human adipose‐derived mesenchymal stem cells (MSCs). (**A**) Morphology of MSCs. Human adipose‐derived MSCs displayed spindle‐shaped morphology in normal 2D culture. Morphologies of 3D spheroids in drops and 3D spheroids‐derived cells after injection onto culture plates were also shown (bar = 100 μm); (**B**) Immunophenotype of MSCs. The immunophenotype of human adipose‐derived MSCs was analyzed by flow cytometry. Most cells expressed CD105 and CD90, but were CD45‐ and CD34‐negative. (**C**) Multipotency of MSCs. Human adipose‐derived MSCs could differentiate into adipogenic and osteogenic lineages when cultured in differentiation medium (bar = 100 μm).

**Figure 2:**
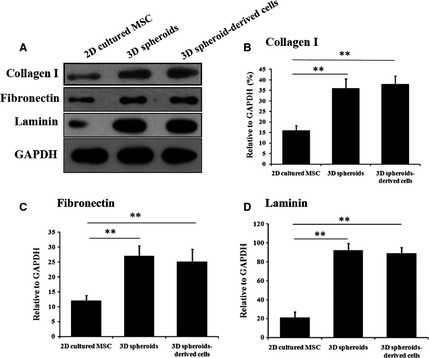
Secretion of extracellular matrix (ECM) proteins by human adipose‐derived mesenchymal stem cells (MSCs) under different culture conditions. (**A**) Western blotting analysis of ECM proteins. ECM proteins (collagen I, fibronectin and laminin) produced by 2D cultured MSCs, 3D spheroids of MSCs and 3D spheroids‐derived cells, were analyzed by Western blotting. GAPDH was the internal control. Band intensities were quantified and normalized to the internal control. (**B**) Statistical analysis of collagen I levels. (**C**) Statistical analysis of fibronectin levels. (**D**) Statistical analysis of laminin levels (**P < 0.01).

Human adipose‐derived MSCs were 2D cultured or cultured as 3D spheroids (Fig. 1A). The immunophenotype of MSCs was analyzed by flow cytometry (Fig. 1B), and the multipotency of MSCs was verified by osteogenic and adipogenic differentiation (Fig. 1C). It was reported that majority of transplanted MSCs died of anoikis in severe microenvironments such as ischemia, hypoxia and active oxygen, because they could not build timely connections to surrounding tissues through ECM proteins 36. Therefore, we first examined the production of ECM proteins by 3D spheroids of MSCs, as well as their anti‐oxidative and anti‐apoptotic capabilities. Western blotting analysis indicated the expression levels of ECM proteins, including collagen I, fibronectin and laminin, were much higher in 3D spheroids and 3D spheroid‐derived cells, compared with 2D cultured cells (Fig. 2).

### Expression of anti‐oxidative proteins and apoptosis‐related proteins by MSCs after H_2_O_2_ treatment

**Figure 3:**
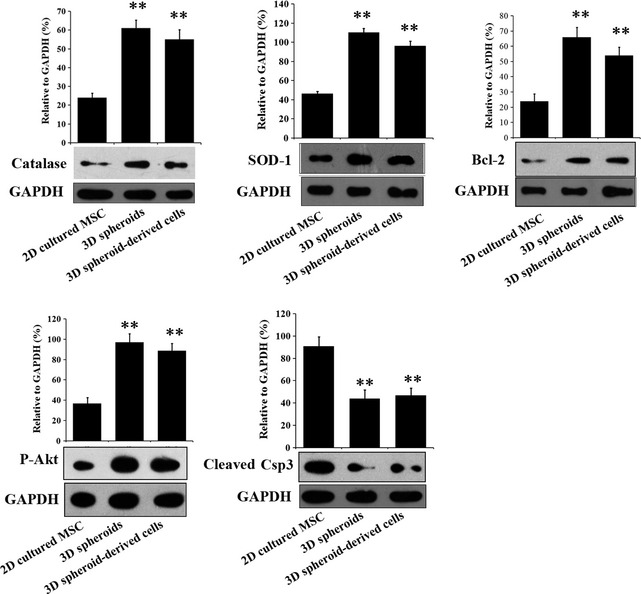
Expression levels of anti‐oxidative proteins and apoptosis‐related proteins by human adipose‐derived mesenchymal stem cells (MSCs) under H_2_O_2_ stimulation. 2D cultured MSCs, 3D spheroids of MSCs and 3D spheroids‐derived cells were treated with 100 μM of H_2_O_2_, respectively. Anti‐oxidative proteins [catalase and superoxide dismutase 1 (SOD‐1)], anti‐apoptotic proteins (Bcl‐2 and p‐Akt), and the apoptotic protein, cleaved caspase3 (Csp3) were analyzed by Western blotting. Band intensities were quantified and normalized to the GAPDH internal control. **P < 0.01 compared with 2D cultured MSCs.

When stimulated by an oxidative condition of H_2_O_2_ (100 μM), 3D spheroids and 3D spheroid‐derived cells had significantly higher expression of the ROS‐scavenging proteins, catalase and SOD‐1, compared with 2D cultured MSCs (Fig. 3). Furthermore, the anti‐apoptotic protein Bcl‐2 and the pro‐survival protein phosphorylated Akt were increased in 3D spheroids and 3D spheroid‐derived cells, while the expression of the cleaved caspase 3 was significantly lower (Fig. 3). These results implied that 3D spheroids might have stronger survival capabilities than 2D cultured MSCs when transplanted into the severe microenvironments of AKI.

### Secretion of beneficial cytokines by MSCs *in vitro*

**Figure 4:**
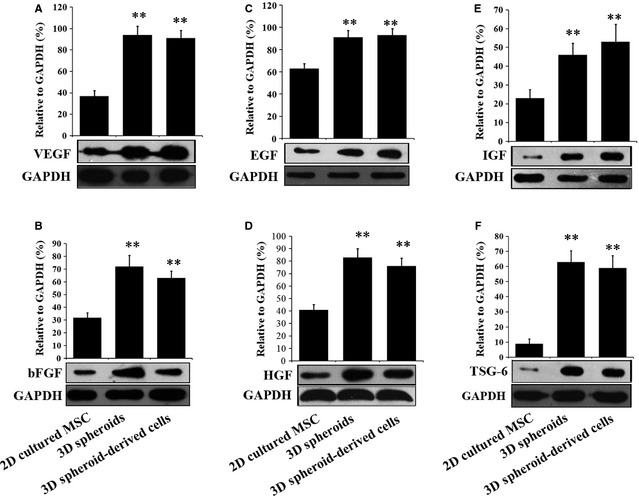
Paracrine secretion of human adipose‐derived mesenchymal stem cells (MSCs) under different culture conditions. The paracrine secretion of cytokines by 2D cultured MSCs, 3D spheroids of MSCs and 3D spheroids‐derived cells was investigated by Western blotting. The levels of VEGF (**A**), basic fibroblast growth factor (bFGF) (**B**), epidermal growth factor (EGF) (**C**), hepatocyte growth factor (HGF) (**D**), insulin‐like growth factor (IGF) (**E**) and tumour necrosis factor‐alpha (TNF‐α) stimulated gene/protein 6 (TSG‐6) (**F**) was determined. Band intensities were quantified and normalized to the GAPDH internal control. **P < 0.01 compared with 2D cultured MSCs.

The low paracrine secretion of MSCs under normal culture conditions also limits their therapeutic effects. Therefore, we next investigated whether 3D spheroids of MSCs exhibited enhanced paracrine secretions than 2D cultured cells. The secretions of the angiogenic factors, VEGF (Fig. 4A) and bFGF (Fig. 4B), were significantly higher in 3D spheroids and 3D spheroid‐derived cells than 2D cultured MSCs, which is consistent with previous studies 29. Furthermore, 3D spheroids and 3D spheroid‐derived cells produced increased levels of anti‐apoptotic factors, including epidermal growth factor (EGF, Fig. 4C) and HGF (Fig. 4D), as well as the anti‐oxidative IGF (Fig. 4E). Finally, the expression of the anti‐inflammatory protein TSG‐6 was also higher in 3D spheroids and 3D spheroid‐derived cells (Fig. 4F), as reported by Bartosh and colleagues 28. Altogether, these results indicated that the paracrine effects were enhanced in 3D spheroids of MSCs, which was proposed to increase their therapeutic benefits.

### Survival and paracrine effects of MSCs *in vivo*

**Figure 5:**
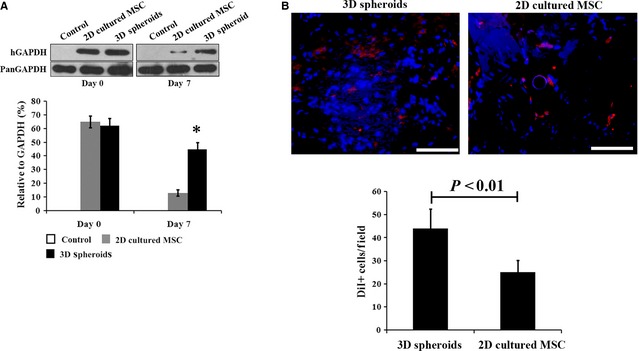
Cell survival after injection in injured kidney. (**A**) Western blotting analysis of human specific GAPDH. The transplanted human adipose‐derived mesenchymal stem cells (MSCs) were detected by Western blotting using human specific antibodies at days 0 and 7 after surgery, which provided indirect information about the survival of MSCs in the kidney. *P < 0.01 compared with 2D cultured MSCs. (**B**) DiIpositive cells detected from kidney sections 1 week after cell injection (bar = 50 μm).

**Figure 6:**
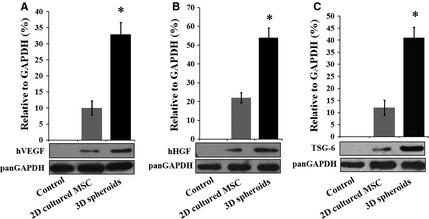
Paracrine secretion of mesenchymal stem cells (MSCs) after injection in injured kidney. Human‐specific therapeutic cytokines secreted by transplanted MSCs, including hVEGF (**A**), hHGF (**B**) and hTSG‐6 (**C**), were determined by Western blotting 7 days after surgery. Band intensities were quantified and normalized to the GAPDH internal control. *P < 0.01 compared with 2D cultured MSCs.

2D cultured MSCs and 3D spheroid‐derived cells were injected into model rats of I/R‐induced AKI, with physiological saline as the control. Survival of transplanted MSCs in the damaged kidney was examined by Western blotting using a human‐specific GAPDH antibody as previously reported 37. The survival rate of 3D spheroids was significantly higher at day 7 after injection than that of 2D cultured cells (Fig. 5A). The survival of MSCs was further confirmed by DiI staining. As shown in Figure 5B, significantly more DiI positive cells were detected from animals receiving 3D spheroids injection at 1 week. In addition, the transplanted 3D spheroids exhibited higher paracrine secretions than 2D cultured cells, as represented by VEGF (Fig. 6A), HGF (Fig. 6B) and TSG‐6 (Fig. 6C). Therefore, our results showed that transplanted 3D spheroids had enhanced survival and paracrine effects than 2D cultured MSCs, which was consistent with *in vitro* experiments.

### Renal function

**Figure 7:**
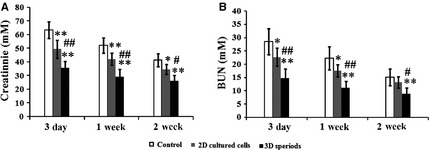
Renal function after surgery. Blood was taken 3 days, 1 week and 2 weeks after surgery (*n* = 5 for each group). Levels of serum creatinine (**A**) and blood urea nitrogen (BUN) (**B**) were measured. *P < 0.05 compared with control; **P < 0.01 compared with control; ^#^ *P* < 0.05 compared with 2D cultured mesenchymal stem cell (MSC) group; ^##^ *P* < 0.01 compared with 2D cultured MSC group.

We next tested whether injected 3D spheroids were more beneficial in ameliorating renal function compared with 2D cultured MSCs. At 3 days, 1 week and 2 weeks after MSC transplantation, the concentrations of serum creatinine and BUN in each group were measured to evaluate the renal function. Both creatinine and BUN levels were significantly lowered in MSC injection groups at each time‐point, including 2D cultured cells and 3D spheroids (Fig. 7A and B). Especially, injection of 3D spheroids was more effective than 2D cultured cells (Fig. 7A and B). In the following studies, we further investigated the mechanism for renal function improvement in several aspects, including tissue apoptosis, tissue damage and vascularization.

### Tissue apoptosis

**Figure 8:**
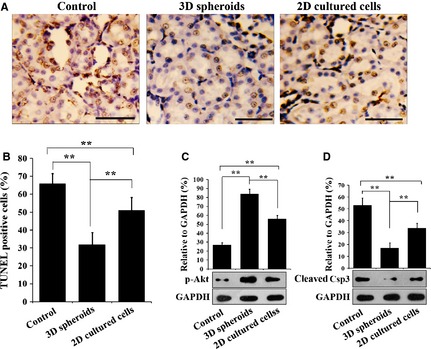
Apoptosis in injured kidney tissues. (**A**) TUNEL staining. Three days after surgery, the kidneys were obtained and fixed in 4% paraformaldehyde (*n* = 5 for each group). Four micrometre paraffin‐embedded sections were prepared. The TUNEL assay was performed using the TUNEL Staining Kit. (bar = 25 μm) (B) Statistical analysis of terminal deoxynucleotidyl transferase dUTP nick‐end labelling (TUNEL)‐positive cells. Positive‐stained cells were counted under microscopy. For each animal, 5 discontinuous sections were observed and 5 random fields were selected on each section. (**C** and **D**) Western blotting analysis of p‐Akt and caspase 3. The levels of p‐Akt and caspase 3 (Csp3) in the kidney were determined by Western blotting, with GAPDH as the internal control (**P < 0.01).

Three days after MSC injection, TUNEL staining was performed to determine apoptosis in the I/R kidney tissues. After TUNEL staining, kidney sections were observed under light microscopy (Fig. 8A), and positively stained cells were quantified (Fig. 8B). Injection of 2D cultured MSCs decreased the apoptotic cells significantly, compared with the saline control (*P* < 0.01), while injection of 3D spheroids further decreased the apoptotic cells (*P* < 0.01). Western blotting analysis of the apoptotic protein caspase 3 (Csp3) and the pro‐survival protein Akt provided consistent results with TUNEL staining. Compared with the control, the expression of cleaved Csp3 in the I/R kidney tissues was significantly decreased in 2D cultured MSC injection group (*P* < 0.01, Fig. 8C), while the level of phosphorylated Akt was significantly raised (*P* < 0.01, Fig. 8D). More importantly, injection of 3D spheroids further reduced the expression of cleaved Csp3 and further promoted Akt phosphorylation, compared with 2D cultured cells (*P* < 0.01, Fig. 8C and D). These results indicated that 3D spheroids protect the I/R kidney against apoptosis more effectively than 2D cultured cells.

### Tissue damage

**Figure 9:**
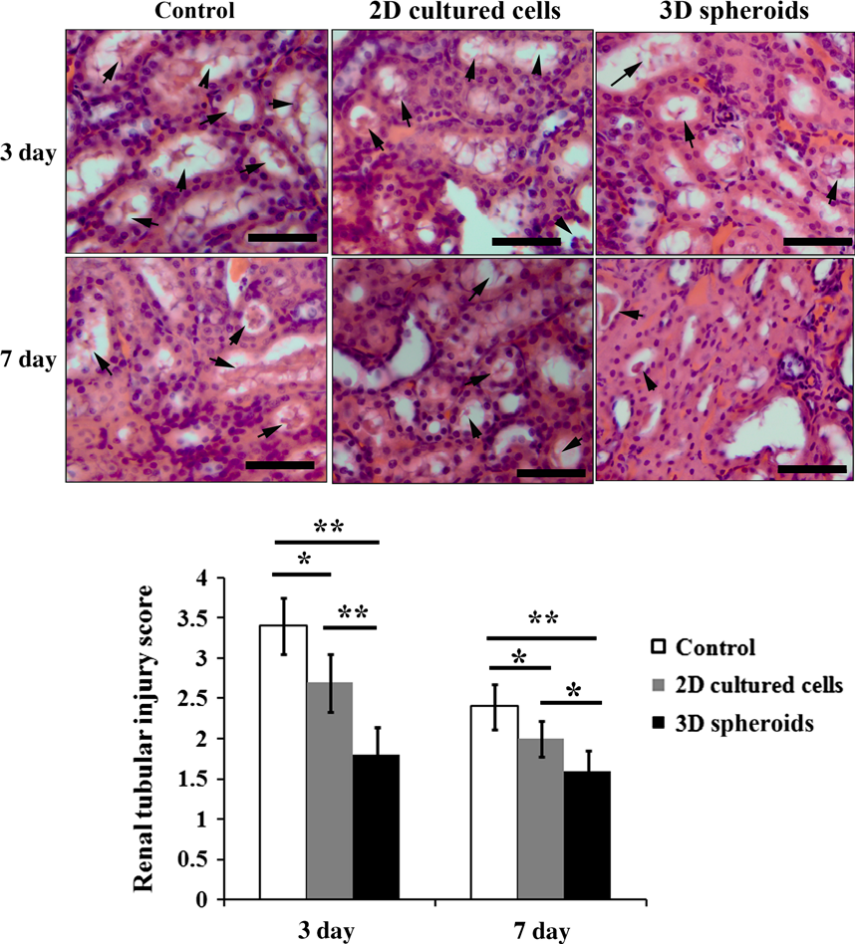
Histological evaluation of tissue damage. At days 3 and 7 after surgery, the kidneys were obtained and fixed in 4% paraformaldehyde (*n* = 5 for each group). To determine tissue damage, haematoxylin and eosin staining was performed on paraffin‐embedded sections of days 3 and 7. The arrows indicated tubular cell necrosis. It can be observed that there was less tubular cell necrosis in mesenchymal stem cell treated groups than the saline control, especially for 3D spheroids (*P < 0.05; **P < 0.01; bar = 50 μm).

Three days and 1 week after cell injection, kidney tissue specimens from each group were obtained, and subjected to haematoxylin and eosin staining. Compared with the saline control, injection of 2D cultured cells significantly attenuated I/R‐induced renal injury, as indicated by less tubular cell necrosis, less loss of brush border and lower grade of tubular dilatation (Fig. 9). Further improvement of histological morphology could be observed in 3D spheroids group compared with that in 2D cultured cells group (Fig. 9). Quantitative analysis showed that histological scores for renal injury in 2D cultured cells injection group were significantly lower than that in the control group (*P* < 0.05, Fig. 9), while the histological scores of renal injury in 3D spheroids group were further lower than 2D cultured cells group (*P* < 0.01 for day 3 and *P* < 0.05 for day 7, Fig. 9).

### Vascularization

**Figure 10:**
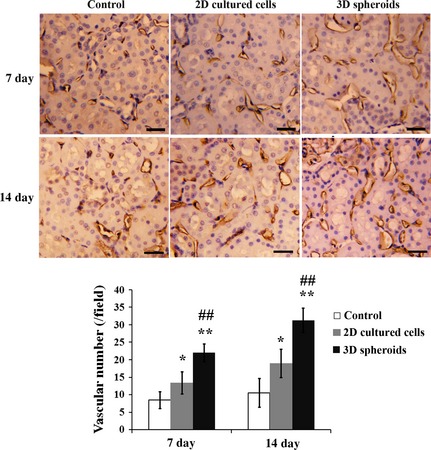
Vascular densities in injured renal tissues. At days 7 and 14 after surgery, the kidneys were obtained and fixed in 4% paraformaldehyde (*n* = 5 for each group). Immunostaining with anti‐vWF antibodies (Sigma‐Aldrich) was carried out on paraffin‐embedded sections of days 7 and 14 to determine the neovascularization in injured renal tissues. Positively‐stained vessels were quantified, and the vascular density was expressed as the number of capillary vessels per field. *P < 0.05 compared with control; **P < 0.01 compared with control; ^##^ *P* < 0.01 compared with 2D cultured mesenchymal stem cell group (bar = 25 μm).

Finally, vascularization was determined at days 7 and 14 after MSC transplantation. As shown in Figure 10, injection of 2D cultured cells significantly increased the vascular density in ischemic kidney tissues compared with the saline control at both time‐points (*P* < 0.05). Furthermore, the vascular density was even higher in 3D spheroids group than 2D cultured cells group (*P* < 0.01).

## Discussion

Mesenchymal stem cell‐based therapy is a promising strategy for AKI treatment. Paracrine secretion is proposed as an important mechanism for stem cell‐based tissue repair 17, 18. However, the therapeutic benefits of MSCs for AKI were limited. There are mainly two reasons: first, the paracrine effects of MSCs under normal culture conditions are quite low; second, transplanted MSCs may die in the severe microenvironments before they can release beneficial cytokines. 3D culture strategy has been reported to increase the therapeutic potentials of MSCs 27, 28. In the present study, the therapeutic effects of 2D cultured MSCs and 3D spheroids of MSCs for AKI were compared. We showed that transplanted 3D spheroids had enhanced paracrine effects *in vivo* and were more effective for AKI treatment.

The 3D culture method promotes cell‐cell and cell‐ECM interactions, and is supposed to provide a cellular environment more consistent with that *in vivo* 27. Our results indicated that 3D spheroids of MSCs produced higher levels of ECM proteins (including collagen I, fibronectin and laminin), and exhibited stronger anti‐apoptotic and anti‐oxidative capacities under ROS stimulation *in vitro*. These results implied that 3D spheroids would have improved survival than 2D cultured cells *in vivo*. The increased survival rate of 3D spheroids was confirmed by Western blotting using human‐specific antibodies, which provided an indirect estimation of the survived cells 37. We further labelled MSCs with DiI for histological analysis to acquire the direct evidence for the retention of injected MSCs in recipients. Besides, MSCs can be stably transfected with firefly luciferase or green fluorescent protein reporter gene, which allows for *in vivo* bioluminescence or fluorescence imaging and tracking of transplanted cells.

The 3D culture strategy has been reported to promote the paracrine secretion of therapeutic cytokines by MSCs, including the anti‐inflammatory protein TSG‐6 28 and the angiogenic factors (VEGF and bFGF) 29. Our studies showed that 3D spheroids also produced higher levels of the anti‐apoptotic factors (EGF and HGF) and the anti‐oxidative factor IGF than 2D cultured MSCs. The paracrine action of transplanted MSCs was supposed to be an important mechanism of stem cell‐based therapy 16. Consistent with the enhanced paracrine secretions, 3D spheroids were more effective in protecting the damaged kidney against apoptosis, reducing tissue damage, promoting vascularization and ameliorating renal function than 2D cultured cells.

Several other strategies have been studied to enhance the therapeutic benefits of MSCs, including: (*i*) gene modification 16, 23, 24, (*ii*) co‐transplantation with adjuvant 33, 34 and (*iii*) hypoxia preconditioning 19, 20, 21, 22. These strategies either increase the survival of MSCs in ischemic tissues, or enhance their paracrine effects. Akt‐modified MSCs were reported to have enhanced paracrine action 16, while overexpression of the antiapoptotic Bcl‐2 inhibited apoptosis of MSCs and increased their engraftment 23. However, gene modification is limited in practical application because of the risk to activate oncogenes. Co‐transplantation with adjuvant such as chitosan hydrogel facilitates the retention and survival of stem cells, but fails to stimulate their paracrine secretions. Similar to the 3D culture strategy, hypoxia preconditioning not only increased the survival of transplanted MSCs, but also activated the expression of pro‐survival and angiogenic factors 19, 20, 21, 22.

In conclusion, our studies showed that transplanted 3D spheroids of human adipose‐derived MSCs had higher survival rate and enhanced paracrine secretions compared with 2D cultured cells. Importantly, the 3D culture strategy improved the therapeutic effects of MSCs for AKI, characterized by reduced cell apoptosis, less tissue damage, increased vascularization and improved renal function.

## Conflicts of interest

The authors have declared that no competing interests exist.

## References

[1] Thomas ME , Blaine C , Dawnay A , *et al* The definition of acute kidney injury and its use in practice. Kidney Int. 2015; 87: 62–73. 25317932 10.1038/ki.2014.328

[2] Bonventre JV , Weinberg JM . Recent advances in the pathophysiology of ischemic acute renal failure. J Am Soc Nephrol. 2003; 14: 2199–210. 12874476 10.1097/01.asn.0000079785.13922.f6

[3] Lattanzio MR , Kopyt NP . Acute kidney injury: new concepts in definition, diagnosis, pathophysiology, and treatment. J Am Osteopath Assoc. 2009; 109: 13–9. 19193820

[4] Kellum JA , Bellomo R , Ronco C . Definition and classification of acute kidney injury. Nephron Clin Pract. 2008; 109: c182–7. 18802365 10.1159/000142926

[5] Morigi M , Imberti B , Zoja C , *et al* Mesenchymal stem cells are renotropic, helping to repair the kidney and improve function in acute renal failure. J Am Soc Nephrol. 2004; 15: 1794–804. 15213267 10.1097/01.asn.0000128974.07460.34

[6] Lange C , Togel F , Ittrich H , *et al* Administered mesenchymal stem cells enhance recovery from ischemia/reperfusion‐induced acute renal failure in rats. Kidney Int. 2005; 68: 1613–7. 16164638 10.1111/j.1523-1755.2005.00573.x

[7] Lazzeri E , Crescioli C , Ronconi E , *et al* Regenerative potential of embryonic renal multipotent progenitors in acute renal failure. J Am Soc Nephrol. 2007; 18: 3128–38. 17978305 10.1681/ASN.2007020210

[8] Rota C , Imberti B , Pozzobon M , *et al* Human amniotic fluid stem cell preconditioning improves their regenerative potential. Stem Cells Dev. 2011; 21: 1911–23. 22066606 10.1089/scd.2011.0333 PMC3396139

[9] Li L , Black R , Ma Z , *et al* Use of mouse hematopoietic stem and progenitor cells to treat acute kidney injury. Am J Physiol Renal Physiol. 2011; 302: F9–19. 21937606 10.1152/ajprenal.00377.2011 PMC3251347

[10] Morigi M , Introna M , Imberti B , *et al* Human bone marrow mesenchymal stem cells accelerate recovery of acute renal injury and prolong survival in mice. Stem Cells. 2008; 26: 2075–82. 18499895 10.1634/stemcells.2007-0795

[11] Morigi M , Rota C , Montemurro T , *et al* Life‐sparing effect of human cord blood‐mesenchymal stem cells in experimental acute kidney injury. Stem Cells. 2010; 28: 513–22. 20049901 10.1002/stem.293

[12] Qi S , Wu D . Bone marrow‐derived mesenchymal stem cells protect against cisplatin‐induced acute kidney injury in rats by inhibiting cell apoptosis. Int J Mol Med. 2013; 32: 1262–72. 24126885 10.3892/ijmm.2013.1517 PMC3829764

[13] Togel F , Hu Z , Weiss K , *et al* Administered mesenchymal stem cells protect against ischemic acute renal failure through differentiation‐independent mechanisms. Am J Physiol Renal Physiol. 2005; 289: F31–42. 15713913 10.1152/ajprenal.00007.2005

[14] Zhuo W , Liao L , Xu T , *et al* Mesenchymal stem cells ameliorate ischemia‐reperfusion‐induced renal dysfunction by improving the antioxidant/oxidant balance in the ischemic kidney. Urol Int. 2010; 86: 191–6. 20881358 10.1159/000319366

[15] Chen YT , Sun CK , Lin YC , *et al* Adipose‐derived mesenchymal stem cell protects kidneys against ischemia‐reperfusion injury through suppressing oxidative stress and inflammatory reaction. J Transl Med. 2011; 9: 51. 21545725 10.1186/1479-5876-9-51 PMC3112438

[16] Gnecchi M , He H , Liang OD , *et al* Paracrine action accounts for marked protection of ischemic heart by akt‐modified mesenchymal stem cells. Nat Med. 2005; 11: 367–8. 15812508 10.1038/nm0405-367

[17] Rubina K , Kalinina N , Efimenko A , *et al* Adipose stromal cells stimulate angiogenesis *via* promoting progenitor cell differentiation, secretion of angiogenic factors, and enhancing vessel maturation. Tissue Eng Part A. 2009; 15: 2039–50. 19368510 10.1089/ten.tea.2008.0359

[18] Verseijden F , Jahr H , Posthumus‐van Sluijs SJ , *et al* Angiogenic capacity of human adipose‐derived stromal cells during adipogenic differentiation: an *in vitro* study. Tissue Eng Part A. 2009; 15: 445–52. 18652540 10.1089/ten.tea.2007.0429

[19] Hu X , Yu SP , Fraser JL , *et al* Transplantation of hypoxia‐preconditioned mesenchymal stem cells improves infarcted heart function *via* enhanced survival of implanted cells and angiogenesis. J Thorac Cardiovasc Surg. 2008; 135: 799–808. 18374759 10.1016/j.jtcvs.2007.07.071

[20] Park BS , Kim WS , Choi JS , *et al* Hair growth stimulated by conditioned medium of adipose‐derived stem cells is enhanced by hypoxia: evidence of increased growth factor secretion. Biomed Res. 2010; 31: 27–34. 20203417 10.2220/biomedres.31.27

[21] Wei L , Fraser JL , Lu ZY , *et al* Transplantation of hypoxia preconditioned bone marrow mesenchymal stem cells enhances angiogenesis and neurogenesis after cerebral ischemia in rats. Neurobiol Dis. 2012; 46: 635–45. 22426403 10.1016/j.nbd.2012.03.002 PMC3353023

[22] Liu L , Gao J , Yuan Y , *et al* Hypoxia preconditioned human adipose derived mesenchymal stem cells enhance angiogenic potential *via* secretion of increased vegf and bfgf. Cell Biol Int. 2013; 37: 551–60. 23505143 10.1002/cbin.10097

[23] Li W , Ma N , Ong LL , *et al* Bcl‐2 engineered mscs inhibited apoptosis and improved heart function. Stem Cells. 2007; 25: 2118–27. 17478584 10.1634/stemcells.2006-0771

[24] Liu N , Patzak A , Zhang J . Cxcr4‐overexpressing bone marrow‐derived mesenchymal stem cells improve repair of acute kidney injury. Am J Physiol Renal Physiol. 2013; 305: F1064–73. 23884141 10.1152/ajprenal.00178.2013

[25] Arufe MC , De la Fuente A , Fuentes‐Boquete I , *et al* Differentiation of synovial cd‐105(+) human mesenchymal stem cells into chondrocyte‐like cells through spheroid formation. J Cell Biochem. 2009; 108: 145–55. 19544399 10.1002/jcb.22238

[26] Wang W , Itaka K , Ohba S , *et al* 3d spheroid culture system on micropatterned substrates for improved differentiation efficiency of multipotent mesenchymal stem cells. Biomaterials. 2009; 30: 2705–15. 19215979 10.1016/j.biomaterials.2009.01.030

[27] Frith JE , Thomson B , Genever PG . Dynamic three‐dimensional culture methods enhance mesenchymal stem cell properties and increase therapeutic potential. Tissue Eng Part C Methods. 2009; 16: 735–49. 19811095 10.1089/ten.TEC.2009.0432

[28] Bartosh TJ , Ylostalo JH , Mohammadipoor A , *et al* Aggregation of human mesenchymal stromal cells (mscs) into 3d spheroids enhances their antiinflammatory properties. Proc Natl Acad Sci USA. 2010; 107: 13724–9. 20643923 10.1073/pnas.1008117107 PMC2922230

[29] Potapova IA , Gaudette GR , Brink PR , *et al* Mesenchymal stem cells support migration, extracellular matrix invasion, proliferation, and survival of endothelial cells *in vitro* . Stem Cells. 2007; 25: 1761–8. 17395769 10.1634/stemcells.2007-0022

[30] Potapova IA , Brink PR , Cohen IS , *et al* Culturing of human mesenchymal stem cells as three‐dimensional aggregates induces functional expression of cxcr4 that regulates adhesion to endothelial cells. J Biol Chem. 2008; 283: 13100–7. 18334485 10.1074/jbc.M800184200 PMC2442325

[31] Wang CC , Chen CH , Hwang SM , *et al* Spherically symmetric mesenchymal stromal cell bodies inherent with endogenous extracellular matrices for cellular cardiomyoplasty. Stem Cells. 2009; 27: 724–32. 19259939 10.1634/stemcells.2008-0944

[32] Zuk PA , Zhu M , Ashjian P , *et al* Human adipose tissue is a source of multipotent stem cells. Mol Biol Cell. 2002; 13: 4279–95. 12475952 10.1091/mbc.E02-02-0105 PMC138633

[33] Liu Z , Wang H , Wang Y , *et al* The influence of chitosan hydrogel on stem cell engraftment, survival and homing in the ischemic myocardial microenvironment. Biomaterials. 2012; 33: 3093–106. 22265788 10.1016/j.biomaterials.2011.12.044

[34] Gao J , Liu R , Wu J , *et al* The use of chitosan based hydrogel for enhancing the therapeutic benefits of adipose‐derived mscs for acute kidney injury. Biomaterials. 2012; 33: 3673–81. 22361096 10.1016/j.biomaterials.2012.01.061

[35] Dai W , Hale SL , Martin BJ , *et al* Allogeneic mesenchymal stem cell transplantation in postinfarcted rat myocardium: short‐ and long‐term effects. Circulation. 2005; 112: 214–23. 15998673 10.1161/CIRCULATIONAHA.104.527937

[36] Lee S , Choi E , Cha MJ , *et al* Cell adhesion and long‐term survival of transplanted mesenchymal stem cells: a prerequisite for cell therapy. Oxid Med Cell Longev. 2015; 2015: 632902. 25722795 10.1155/2015/632902 PMC4333334

[37] Chimenti I , Smith RR , Li TS , *et al* Relative roles of direct regeneration *versus* paracrine effects of human cardiosphere‐derived cells transplanted into infarcted mice. Circ Res. 2010; 106: 971–80. 20110532 10.1161/CIRCRESAHA.109.210682 PMC4317351

